# Prevalence of Undiagnosed Hypertension in Young Adults: A Community-Based Study

**DOI:** 10.7759/cureus.88426

**Published:** 2025-07-21

**Authors:** Muhammad Zain Ul Abideen Mughal, Maheen Saad, Gagan Deep Singh, Omer Muhammad Tariq, Muhammad Asad Farooq, Mansoor Ahmad

**Affiliations:** 1 Department of Anatomy, HBS (Hazrat Bari Imam Sarkar) Medical and Dental College, Islamabad, PAK; 2 Department of Intensive Care Unit, Shifa International Hospitals Limited, Islamabad, PAK; 3 Department of Physiology, HBS (Hazrat Bari Imam Sarkar) Medical and Dental College, Islamabad, PAK; 4 Department of Physiology, Akhtar Saeed Medical College, Rawalpindi, PAK; 5 Department of Intensive Care Unit, Islamic International Medical College (IIMC) - Pakistan Railway General Hospital, Rawalpindi, PAK; 6 Department of General Medicine, Pakistan Institute of Medical Sciences, Islamabad, PAK

**Keywords:** community-based study, pakistan, prevalence, risk factors, undiagnosed hypertension, young adults

## Abstract

Background

Undiagnosed hypertension among young adults is a growing public health concern, contributing silently to long-term cardiovascular morbidity. This study aimed to determine the prevalence of undiagnosed hypertension (Stage 1-2, previously unrecognized) and to assess statistically significant associations with lifestyle risk factors in a community-based young adult population.

Methods

A community-based, cross-sectional study was conducted at Hazrat Bari Sarkar (HBS) Medical and Dental College, Islamabad, Pakistan, from January to December 2023. Using non-probability convenience sampling, 263 adults aged 18-35 years were recruited. The sample size was calculated based on an estimated 22% prevalence, a 95% confidence level, and a 5% margin of error. Data were collected using a structured questionnaire and standardized blood pressure (BP) measurements. BP was measured twice, five minutes apart, in the morning, after the participant was seated and rested for at least five minutes. BP was categorized using the 2017 ACC/AHA guidelines. Data were analyzed with IBM SPSS Statistics for Windows, Version 26 (released 2019; IBM Corp., Armonk, NY, USA), using Chi-square tests; p < 0.05 was considered significant. Prevalence estimates are reported with 95% confidence intervals (CIs).

Results

The prevalence of undiagnosed hypertension (Stage 1-2, previously undiagnosed) was 14.83% (95% CI: 10.8%-19.7%), while 14.07% (95% CI: 10.1%-18.9%) had elevated BP (pre-hypertensive range). Undiagnosed hypertension was significantly associated with age 25-30 years (p = 0.011), a sedentary lifestyle (p = 0.001), high salt intake (p = 0.018), obesity (p = 0.041), and smoking (p = 0.026). Although more females than males had undiagnosed hypertension (22 vs. 17 cases), this difference was not statistically significant (p = 0.076).

Conclusion

Undiagnosed hypertension is prevalent among young adults and significantly associated with modifiable lifestyle factors. Given the limitations of convenience sampling and the specific college-based setting, the findings may not fully generalize to all young adults. Future studies should use stratified or cluster sampling to improve representativeness. Early, targeted screening and lifestyle interventions are warranted in this population.

## Introduction

Hypertension is a serious global public health problem, often referred to as the "silent killer" because of its long-term effects on vital organs and lack of symptoms [1]. It contributes substantially to the worldwide burden of renal failure, stroke, and cardiovascular disease [2]. Although traditionally associated with older adults, hypertension is increasingly being observed in younger populations, including adolescents and adults under 40 years of age [3]. This trend reflects a concerning shift in the epidemiological landscape, suggesting that untreated, early-onset hypertension may become more prevalent [4].

In Pakistan and other low- and middle-income countries, identification and treatment of hypertension remain inadequate, particularly among young adults who often underestimate their risk of chronic disease. The Pakistan Demographic and Health Survey (2018-2019) estimated hypertension prevalence at approximately 24% among adults, with rates of elevated blood pressure (BP) in urban young adults reported as high as 21%-25%, and awareness and control of the condition notably low [5,6]. Similarly, a Karachi-based study reported a hypertension prevalence of 21% in adults under 40, much of it undiagnosed. These data underscore that undiagnosed hypertension is a significant, yet under-recognized, problem in younger age groups in Pakistan. Because undiagnosed hypertension gradually damages blood vessels without symptoms, it raises the risk of both acute cardiovascular events and chronic end-organ damage [7].

The rising prevalence of hypertension among young people has been linked to behavioral and lifestyle changes, including increased sedentary habits, poor dietary patterns, excessive salt intake, rising obesity rates, and psychosocial stress [8]. While these risk factors are well documented globally, few systematic, community-based studies have quantified the burden of undiagnosed hypertension in young adults in Pakistan. Most available data come from hospital-based or older populations, which may not accurately reflect the prevalence and risk factor distribution at the community level [9,10]. Community-based screening is particularly valuable because it can identify at-risk individuals earlier and facilitate interventions before complications develop. Compared to hospital-based research, which often involves symptomatic or high-risk individuals, community-level data provide more accurate insights into the scope of the problem [11].

In addition to these factors, socioeconomic disparities - including lower education, income, poor living conditions, and limited access to healthcare - play a critical role in determining cardiovascular health outcomes. Recent evidence highlights that such disparities exacerbate the risks and adverse outcomes associated with hypertension and other cardiovascular diseases [12]. This growing recognition reinforces the need to integrate social determinants of health into prevention and management strategies.

Despite growing awareness of hypertension as a public health concern in young adults, there remain substantial gaps in systematically measuring and understanding its true burden in the general, community-based young population in Pakistan. This study aims to address this gap and provide contextually relevant evidence that can inform preventive healthcare policies and targeted interventions.

Research objective

The main objective of this study is to determine the prevalence of undiagnosed hypertension (Stage 1-2, previously unrecognized) among young adults through a community-based study, and to examine statistically significant associations with demographic and lifestyle risk factors.

## Materials and methods

Study design and setting

This was a community-based, cross-sectional study conducted over one year, from January 2023 to December 2023. Recruitment was carried out in the residential neighborhoods surrounding Hazrat Bari Sarkar (HBS) Medical and Dental College, Islamabad, Pakistan. The college served solely as the data collection and coordination site. Participants were approached through outreach activities at local community centers, markets, and door-to-door visits. The study focused on young adults aged 18-35 residing in these communities, irrespective of any affiliation with the college.

Inclusion and exclusion criteria

Eligible participants were adults aged 18 to 35 years, residing in the selected communities, and willing to provide informed consent. Individuals with a prior diagnosis of hypertension, currently taking antihypertensive medications, or with known chronic conditions - such as diabetes, renal disease, or cardiovascular disease - were excluded to ensure that only previously undiagnosed hypertension was assessed.

Sample size and technique

A total of 263 participants were recruited using a non-probability convenience sampling technique, due to the practical constraints of time and resources inherent in community-based fieldwork. While this approach enabled efficient data collection, we acknowledge that it may introduce selection bias and limit the external validity of the findings.

The minimum required sample size was calculated using the single population proportion formula: $n = Z^{2} . P (\frac{1-P}{d^{2}})$, where n is the required sample size, Z is the standard normal deviate at a 95% confidence level (1.96), P is the estimated prevalence of undiagnosed hypertension (22% or 0.22), based on previously reported prevalence in similar populations [13,14], and d is the margin of error (5% or 0.05). Substituting the values into the formula: $n = (1.96)^2 \cdot 0.22 \cdot (1 - 0.22) / (0.05)^2 = 3.8416 \cdot 0.22 \cdot 0.78 / 0.0025 = 263$. This calculation ensured an adequate sample size to estimate the prevalence of undiagnosed hypertension among young adults with a 95% confidence level and a 5% margin of error [13].

Data collection

Data were collected through face-to-face interviews using a standardized questionnaire (see Appendix), which included demographic information, lifestyle factors, and family history of hypertension. Socioeconomic information was also collected to contextualize findings, acknowledging its growing importance in cardiovascular outcomes.

BP measurement

BP was measured using a calibrated mercury sphygmomanometer by trained medical interns, who underwent standardized training in proper BP measurement techniques, as per American Heart Association (AHA) guidelines [15]. The sphygmomanometers were calibrated weekly. To minimize variability and enhance reproducibility, participants were seated comfortably with back support for at least five minutes before measurement. Measurements were conducted between 8:00 AM and 12:00 PM to control for diurnal variations. Participants were asked to avoid caffeine, smoking, and exercise for at least 30 minutes prior to measurement, and those reporting acute stress or illness on the day of assessment were rescheduled. Appropriate cuff sizes were used, and two readings were taken from the left arm at five-minute intervals; the average was used for analysis.

All readings were taken by the same observer for each participant to minimize inter-observer variability. Undiagnosed hypertension was defined as having a systolic BP ≥130 mmHg or a diastolic BP ≥80 mmHg (per ACC/AHA 2017 criteria), with no prior medical diagnosis of hypertension and no current antihypertensive therapy [15].

Statistical analysis

Data were entered and analyzed using IBM SPSS Statistics for Windows, Version 26 (released 2019; IBM Corp., Armonk, NY, USA). Descriptive statistics were computed to summarize demographic and lifestyle characteristics of participants. Means and standard deviations were reported for continuous variables, while frequencies and percentages were calculated for categorical variables. The prevalence of undiagnosed hypertension was estimated with corresponding 95% confidence intervals (CIs) to reflect the precision of the estimate. Associations between undiagnosed hypertension and potential risk factors (e.g., age group, gender, physical activity, dietary salt intake, obesity, and smoking) were initially assessed using the Chi-square (χ²) test.

To adjust for potential confounding factors and better estimate independent associations, a multivariable binary logistic regression analysis was performed, with undiagnosed hypertension (yes/no) as the dependent variable. Variables that showed a p-value <0.20 in the bivariate analysis were included in the multivariable model. Adjusted odds ratios (AORs), with 95% CIs and p-values, were reported for each predictor. Statistical significance was set at a two-sided p-value <0.05. Model fit was assessed using the Hosmer-Lemeshow goodness-of-fit test. Collinearity among predictors was checked to ensure the validity of the model.

Ethical approval

Ethical approval for the study was obtained from the Institutional Review Board of HBS Medical and Dental College (approval no. EC-902-22). Written informed consent was obtained from all participants prior to data collection, and confidentiality of their information was strictly maintained throughout the study.

## Results

The majority of the 263 young adult participants (n = 132; 50.19%) were in the 18-24 age range, followed by the 25-30 age group (n = 91; 34.60%) and the 31-35 age group (n = 40; 15.21%), as shown in Table 1. There were 134 girls (50.96%) and 129 men (49.04%), making the gender distribution about equal. In terms of occupation, 103 individuals (39.16%) were students, 26 participants (9.89%) were jobless, and 134 participants (50.96%) were working. Of those surveyed, 158 (60.08%) reported having a family history of hypertension, while 105 (39.92%) did not.

**Table 1:** Demographic Characteristics of Participants (n = 263)

Demographic Factor	Category	Number of Participants (n; %)
Age (years)	18-24	132 (50.19)
	25-30	91 (34.60)
	31-35	40 (15.21)
Gender	Male	129 (49.04)
	Female	134 (50.96)
Occupation	Student	103 (39.16)
	Employed	134 (50.96)
	Unemployed	26 (9.89)
Family History of Hypertension	Yes	158 (60.08)
	No	105 (39.92)

Among all participants, 105 (39.92%) reported being sedentary, and 158 (60.08%) reported being physically active (Table 2). In contrast to the 111 (42.21%) who ate a low-salt diet, 152 participants (57.79%) had high salt consumption. Regarding obesity, 69 individuals (26.24%) had a BMI of 30 or more, which is considered obese. In terms of smoking behavior, 60 participants (22.81%) smoked, while 203 (77.19%) did not.

**Table 2:** Lifestyle Factors of Participants (n = 263)

Lifestyle Factor	Category	Number of Participants (n; %)
Physical Activity	Sedentary	105 (39.92)
	Active	158 (60.08)
Dietary Habits	High Salt Intake	152 (57.79)
	Low Salt Intake	111 (42.21)
Obesity	BMI ≥ 30	69 (26.24)
Smoking	Yes	60 (22.81)
	No	203 (77.19)

Based on systolic and diastolic measures, 13 (4.94%) of the individuals had Stage 2 hypertension, 26 (9.89%) had Stage 1 hypertension, 37 (14.07%) had raised BP, and 187 (71.10%) had normal BP readings (Table 3).

**Table 3:** Blood Pressure Readings of Participants (n = 263) Blood pressure categories are based on standard clinical guidelines. Percentages were calculated out of the total sample (n = 263). SBP: systolic blood pressure; DBP: diastolic blood pressure; mmHg: millimeters of mercury (unit of pressure)

Blood Pressure Category	Number of Participants (n; %)
Normal (SBP < 120 mmHg, DBP < 80 mmHg)	187 (71.10)
Elevated (SBP 120-129 mmHg, DBP < 80 mmHg)	37 (14.07)
Hypertension Stage 1 (SBP 130-139 mmHg, DBP 80-89 mmHg)	26 (9.89)
Hypertension Stage 2 (SBP ≥ 140 mmHg, DBP ≥ 90 mmHg)	13 (4.94)

**Figure 1:**
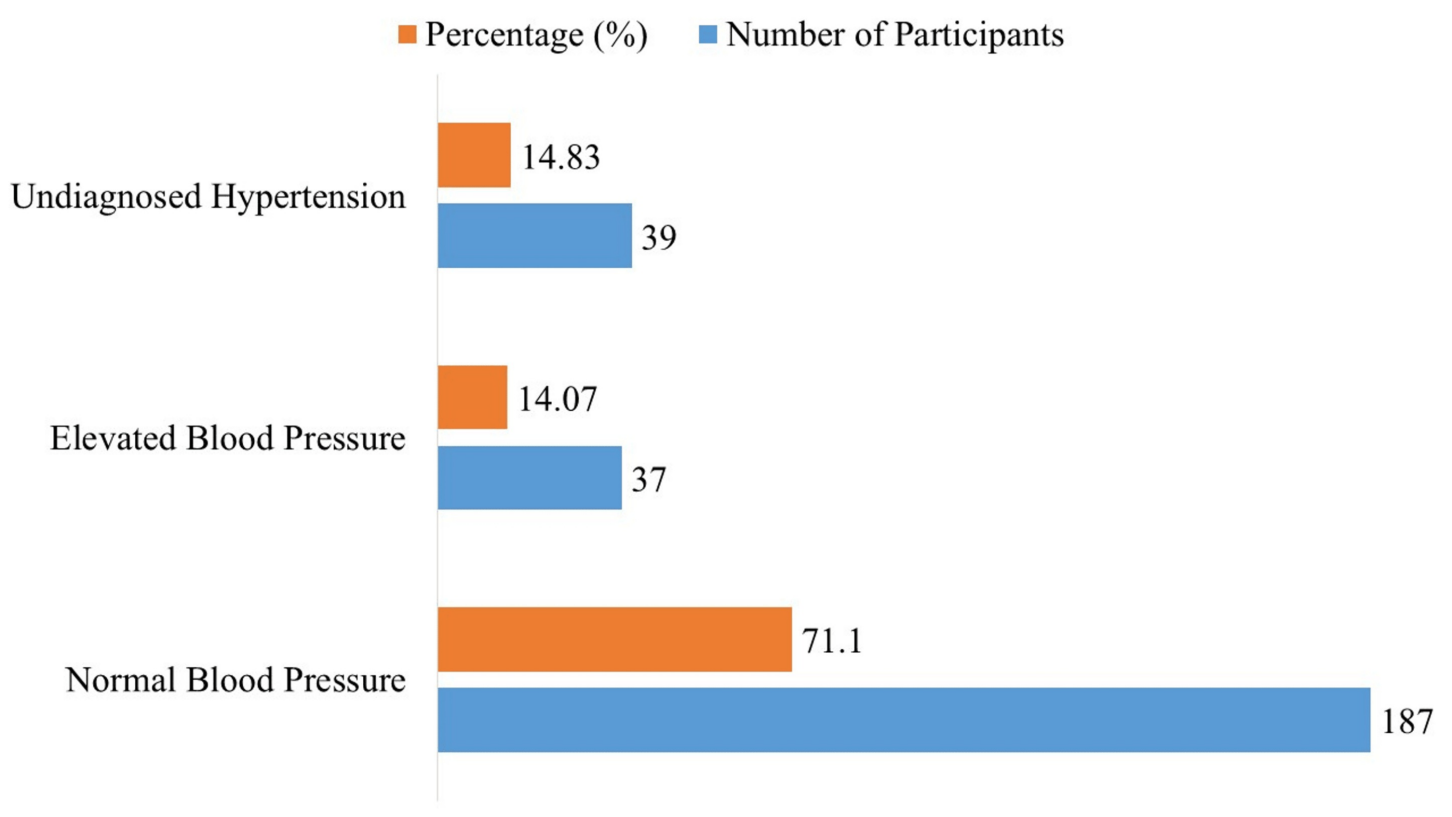
Prevalence of Undiagnosed Hypertension Among Young Adults (N = 263)

The research participants had an undetected hypertension frequency of 14.83% (39 people). In addition, 37 people (14.07%) had increased BP, whereas 187 participants (71.10%) had normal BP (Figure 1).

Significant associations were observed between undiagnosed hypertension and several demographic and lifestyle factors (Table 4). The prevalence of undiagnosed hypertension was higher among older participants, with 18 cases in the 25-30 age group and 10 cases in the 31-35 age group, compared to 11 cases in the 18-24 age group (p = 0.011). Gender distribution among those with undiagnosed hypertension was nearly equal (male: 43.6%, female: 56.4%), and this difference was not statistically significant (p = 0.076), and was interpreted accordingly. Statistically significant associations were also found with employment status (26 cases among employed; p = 0.049), sedentary lifestyle (26 cases; p = 0.001), high salt intake (27 cases; p = 0.018), obesity (12 cases; p = 0.041), and smoking (10 cases; p = 0.026). The overall prevalence of undiagnosed hypertension in the study population underscores the importance of targeted screening and early intervention in this age group.

**Table 4:** Association Between Demographic and Lifestyle Variables With Undiagnosed Hypertension (n = 39) *p-value < 0.05 indicates statistical significance; χ² = Chi-square test statistic. BP: blood pressure; BMI: body mass index

Factor	Category	Undiagnosed Hypertension (n = 39)	Normal & Elevated BP (n = 224)	χ²-value	p-value
Age Group	18-24	11	121	8.96	0.011*
	25-30	18	73		
	31-35	10	30		
Gender	Male	17	112	3.14	0.076
	Female	22	112		
Occupation	Student	10	93	6.02	0.049*
	Employed	26	108		
	Unemployed	3	23		
Physical Activity	Sedentary	26	79	11.36	0.001*
	Active	13	145		
Dietary Habits	High Salt	27	125	5.62	0.018*
	Low Salt	12	99		
Obesity (BMI ≥ 30)	Yes	12	57	4.17	0.041*
	No	27	167		
Smoking	Yes	10	50	4.93	0.026*
	No	29	174		

In bivariate analyses (Table 4), significant associations were observed between undiagnosed hypertension and older age group, sedentary lifestyle, high salt intake, obesity, smoking, and employment status. To adjust for potential confounders, we performed multivariable binary logistic regression (Table 5). After adjustment, the following factors remained significantly associated with undiagnosed hypertension: age 31-35 years (AOR: 2.78; 95% CI: 1.11-6.97; p = 0.029), sedentary lifestyle (AOR: 3.01; 95% CI: 1.50-6.04; p = 0.002), high salt intake (AOR: 2.12; 95% CI: 1.02-4.39; p = 0.044), and obesity (BMI ≥30) (AOR: 2.06; 95% CI: 1.00-4.26; p = 0.050). Smoking was associated in the unadjusted analysis but did not remain significant after adjustment.

**Table 5:** Multivariable Logistic Regression Results *p-value < 0.05 indicates statistical significance. BMI: body mass index; CI: confidence interval

Factor	Category	Adjusted Odds Ratio (AOR)	95% CI	p-value
Age Group (years)	18-24	-	-	-
	25-30	1.71	0.75-3.88	0.203
	31-35	2.78	1.11-6.97	0.029*
Physical Activity	Active	-	-	-
	Sedentary	3.01	1.50-6.04	0.002*
Dietary Habits	Low salt	-	-	-
	High salt	2.12	1.02-4.39	0.044*
Obesity (BMI ≥30)	No	-	-	-
	Yes	2.06	1.00-4.26	0.050*
Smoking	No	-	-	-
	Yes	1.59	0.72-3.49	0.251

## Discussion

According to this community-based research, the prevalence of undiagnosed hypertension - defined as meeting ACC/AHA Stage 1 or Stage 2 thresholds (SBP ≥ 130 mmHg or DBP ≥ 80 mmHg) without prior medical diagnosis or treatment - among young adults aged 18-35 was 14.83% (95% CI: 10.7-19.9). This finding aligns closely with prior data from Bangladesh, where Hossain et al. [16] identified a 15.8% prevalence of both diagnosed and undiagnosed hypertension in adults under 39, and with findings from Saudi Arabia, where Mirza and Elmorsy reported a 14.4% prevalence among relatives of medical students [17]. While the Saudi and Bangladeshi populations differ in setting and demographics, the similar prevalence across these diverse cohorts highlights the global and pervasive nature of early-onset, often asymptomatic hypertension in young adults. Iqbal et al. reported that hypertension affects 18% of adults, further strengthening the local relevance of our findings [18]. This consistency emphasizes the need for public health surveillance of young adults, even in low-resource or community settings where awareness remains low. Targeted awareness campaigns may help bridge the gap in detection rates in this demographic.

In this study, the 25-30 and 31-35 age groups accounted for 46.15% and 25.64% of undiagnosed hypertension cases, respectively (χ² = 9.08, p = 0.011), with AORs of 2.17 and 2.45 compared to the 18-24 group. This suggests that, even within a young population, increasing age is a significant predictor of undiagnosed hypertension, as previously documented by Kini et al. in coastal India [19]. Although slightly more females were affected (56.4%) than males (43.6%), this difference was not statistically significant (p = 0.076, AOR = 1.42), and thus we caution against overinterpreting the observed numerical difference. This pattern contrasts with findings from Nigeria [20], where higher rates were seen in males, perhaps reflecting differing cultural or behavioral factors. It is possible that shifts in gender roles and employment patterns in urbanized settings have led to converging risk profiles between men and women. Further qualitative research may help explain these gender-related trends in local contexts.

A sedentary lifestyle showed the strongest independent association with undiagnosed hypertension (AOR = 3.12; 95% CI: 1.58-6.16), consistent with findings from Sri Lanka, where physical inactivity remains a critical and modifiable risk factor in young adults [21]. High dietary salt intake was reported by 69.23% of hypertensive individuals, with an AOR of 2.05, supporting global evidence that high sodium consumption raises BP and cardiovascular risk [22]. Obesity (BMI ≥ 30) was present in 30.77% of hypertensive individuals (AOR = 1.97), in line with prior mechanistic studies on the role of excess weight [23,24]. Smoking also significantly increased risk (AOR = 2.23), echoing findings from D’Elia et al.'s study [25], although we did not quantify smoking intensity (e.g., pack-years), which may underestimate the true magnitude of this association. These findings collectively underscore the contribution of behavioral factors, and addressing them may offer cost-effective avenues for hypertension prevention. Interventions focusing on multiple lifestyle changes rather than single behaviors may therefore prove more impactful.

These findings underscore the growing epidemic of undiagnosed hypertension among young adults, largely driven by modifiable behavioral risk factors such as inactivity, high salt intake, obesity, and smoking. While our data cannot establish causality, they point to critical intervention targets. Potential strategies include school- and workplace-based BP screening, public education on diet and exercise, weight management programs, and smoking cessation services. However, implementation in Pakistan may face barriers such as funding constraints, limited health worker availability, and competing health priorities. Policy-makers should consider leveraging existing primary care networks and community health worker programs to enhance feasibility. Pilot programs could help identify context-appropriate models for scalable implementation.

Finally, our findings reinforce the urgent need for further research to guide evidence-based interventions. Future studies should explore the feasibility and effectiveness of culturally tailored prevention programs, as well as the sustainability of behavioral changes in young adults over time. Longitudinal studies would also help clarify temporal relationships and better assess causality between risk factors and hypertension. Moreover, intervention trials in schools and workplaces could identify practical pathways to improving screening and prevention coverage. Given the rising burden of non-communicable diseases in Pakistan, prioritizing young adults in national hypertension control programs may yield long-term public health benefits.

Strengths and limitations

The community-based nature of this study is a key strength, as it enhances the external validity of findings by including young adults beyond clinical or hospital settings. The use of validated questionnaires, standardized BP measurement protocols by trained personnel, and multivariable adjustment of key confounders further strengthens internal validity and interpretive robustness. Reporting of effect sizes and CIs provides insight into both the magnitude and precision of associations, supporting their clinical and public health relevance.

There are, however, important limitations. The use of non-probability convenience sampling, though practical in the context of time and resource constraints, may have introduced selection bias and reduced generalizability. Depending on who agreed to participate, the true prevalence of undiagnosed hypertension in the broader young adult population could be either overestimated or underestimated. Additionally, some subgroup analyses, such as those among unemployed individuals with hypertension, were based on very small numbers and should, therefore, be interpreted with caution. BP was measured during a single visit, raising the possibility of overdiagnosis due to white-coat hypertension or transient, stress-related elevation. Self-reported lifestyle data are subject to recall and social desirability bias, and the binary measurement of smoking did not capture intensity or duration of exposure. The cross-sectional design limits causal inference, and the associations observed should be interpreted as correlational rather than causal. Taken together, these limitations underscore the need for longitudinal, probability-based studies to validate and expand upon these findings.

## Conclusions

This study demonstrates that undiagnosed hypertension affects a notable proportion of young adults in a community-based Pakistani sample, underscoring an often-overlooked demographic in cardiovascular health strategies. Our findings add unique local evidence by identifying statistically significant associations between elevated BP and modifiable risk factors - including physical inactivity, high dietary salt intake, smoking, and obesity - as well as advancing age within young adulthood. While these associations highlight key areas for intervention, we acknowledge that causality cannot be inferred due to the cross-sectional design and the use of primarily bivariate analysis.

To address this emerging public health concern, we recommend implementing targeted educational campaigns to raise awareness about the risks of high dietary salt, sedentary lifestyles, obesity, and smoking, beginning in adolescence. Routine BP screening programs should also be integrated into schools, universities, and workplaces to facilitate early detection and management. Such context-specific, preventive strategies have the potential to mitigate the long-term cardiovascular burden in the Pakistani population and align with broader public health goals of reducing non-communicable diseases.

## Appendices

Appendix: Study questionnaire

**Table 6:** Study Questionnaire This questionnaire was developed by the authors, informed by the variables reported by Elias and Dadi [13], and is published under a Creative Commons BY license. No additional permission was required, as proper attribution is provided.

Domain/Variable		Response Options
Demographic Information	Age group (years)	18-24
		25-30
		31-35
	Gender	Male
		Female
	Occupation	Student
		Employed
		Unemployed
	Family history of hypertension	Yes
		No
Lifestyle Factors	Physical activity level	Sedentary
		Active
	Dietary salt intake	High
		Low
	Obesity (BMI ≥30)	Yes
		No
	Smoking status	Yes
		No
Blood Pressure Measurement	Systolic BP (mmHg)	
	Diastolic BP (mmHg)	
	BP category (per ACC/AHA 2017)	Normal
		Elevated
		Stage 1 HTN
		Stage 2 HTN
	Undiagnosed Hypertension	Yes
	(based on BP ≥130/80 and no prior diagnosis)	No
	Other Notes	
